# Supplementary material for: Mesoporous Dual-Semiconductor ZnS/CdS Nanocomposites as Efficient Visible Light Photocatalysts for Hydrogen Generation
Source: Nanomaterials (Basel). 2023 Aug 26;13(17):2426. doi: 10.3390/nano13172426 (PMC10490144; doi:10.3390/nano13172426)
Supplement: Supplementary file 1 [file nanomaterials-13-02426-s001.zip › nanomaterials-2552497-supplementary.pdf]

# Supplementary Materials

# Mesoporous Dual-Semiconductor ZnS/CdS Nanocomposites as Efficient Visible Light Photocatalysts for Hydrogen Generation

**Ioannis Vamvasakis [1,*], Evangelos K. Andreou [1] and Gerasimos S. Armatas [1]**

[1] University of Crete, Department of Materials Science and Technology, 70013 Voutes, Heraklion, Greece.
* Correspondence: j.vamvasakis@gmail.com; Tel.: 0030-2810-545119.

## Supporting Tables

**Table S1.** Chemical composition of the as-prepared mesoporous CdS and ZnS NCAs, along with the mesoporous ZnS/CdS nanocomposites with different ZnS content, according to EDS analysis. The EDS results for the reference materials ZnS/CdS bulk and RNAs with 50 wt.% ZnS content are also shown.

| Sample | Cd (at.%) | Zn (at.%) | S (at.%) | ZnS content[1] (wt.%) |
|---|---|---|---|---|
| CdS NCAs | 50.73 ± 0.25 | 0 | 49.27 ± 0.25 | 0 |
| 10-ZnS/CdS | 43.16 ± 0.49 | 7.08 ± 0.40 | 49.76 ± 0.58 | 9.96 ± 0.23 |
| 30-ZnS/CdS | 27.24 ± 0.15 | 16.93 ± 0.71 | 55.83 ± 0.67 | 29.54 ± 0.44 |
| 50-ZnS/CdS | 17.67 ± 0.19 | 26.58 ± 0.65 | 55.75 ± 0.51 | 50.37 ± 0.20 |
| 70-ZnS/CdS | 9.62 ± 0.19 | 34.76 ± 0.59 | 55.62 ± 0.57 | 70.91 ± 0.03 |
| 90-ZnS/CdS | 4.03 ± 0.17 | 46.81 ± 0.91 | 49.16 ± 0.55 | 88.70 ± 0.13 |
| ZnS NCAs | 0 | 53.65 ± 0.33 | 46.35 ± 0.33 | 100 |
| ZnS/CdS bulk [2] | 21.11 ± 0.51 | 31.31 ± 1.28 | 47.58 ± 0.92 | 50.02 ± 0.24 |
| ZnS/CdS RNAs [3] | 17.93 ± 0.10 | 26.39 ± 0.40 | 55.68 ± 0.39 | 49.82 ± 0.14 |
| 50-ZnS/CdS AC [4] | 15.62 ± 0.26 | 23.42 ± 0.60 | 60.96 ± 0.30 | 50.29 ± 0.10 |
| 50-ZnS/CdS-c [5] | 17.47 ± 0.21 | 31.83 ± 0.53 | 50.70 ± 0.52 | 55.14 ± 0.11 |

[1] As determined on the basis of the EDS Zn and Cd atomic ratios. [2] EDS results for the ZnS/CdS bulk reference sample (50 wt.% ZnS). [3] EDS results for the reference material ZnS/CdS RNAs (RNAs: random NC-aggregates) with 50 wt.% ZnS, synthesized through a template-free oxidative coupling of ZnS and CdS NCs. [4] EDS results for the 50-ZnS/CdS catalyst retrieved after 24 h of photocatalytic stability test (reaction conditions: 20 mg of catalyst dispersed in a 1.4 M $Na_2S$ and 1.0 M $Na_2SO_3$ aqueous electrolyte, irradiated with visible light using a 300 W Xe light with a cut-off filter allowing $\lambda \geq 420$ nm). [5] EDS results for the 50-ZnS/CdS catalyst retrieved after the photocatalytic corrosion experiment with triethanolamine (TEOA) as hole scavenger (reaction conditions: 20 mg of catalyst dispersed in aqueous solution containing 10% v/v TEOA, irradiated for 3 h under visible light using a 300 W Xe light with a cut-off filter allowing $\lambda \geq 420$ nm).

**Table S2.** Comparison of $H_2$-production activities between mesoporous 50-ZnS/CdS and other reported CdZnS-based photocatalysts.

| Sample | Reaction conditions | Light source | $H_2$ evolution rate (mmol $g^{-1}$ $h^{-1}$) | Apparent quantum yield (AQY) | Ref. |
|---|---|---|---|---|---|
| ZnS/$Zn_{1-x}Cd_xS$/CdS | 5 mg catalyst 0.35 M $Na_2S$ & 0.25 M $Na_2SO_3$ (100 mL) | 300 W Xe lamp $\lambda \geq 420$ nm | 106.5 | - | [1] |

| | | | | | |
|---|---|---|---|---|---|
| CdS/ZnS nanorods | 1 mg catalyst<br>0.75 M $Na_2S$ &<br>1.05 M $Na_2SO_3$<br>(20 mL) | 300 W<br>Xe lamp<br>$\lambda \geq 420$ nm | 239<br>(μmol $h^{-1}$ $mg^{-1}$) | 16.8%<br>(420 nm) | [2] |
| 0.5%Ru/$(CdS)_{0.8}$/$(ZnS)_{0.2}$ nanoparticles | 150 mg catalyst<br>0.1 M $Na_2S$ &<br>0.2 M $Na_2SO_3$<br>(150 mL) | 300 W<br>Xe lamp<br>$\lambda \geq 420$ nm | 12.65 | - | [3] |
| Pt-ZnS/CdS<br>2D/1D nanocomposites | 50 mg catalyst<br>~10% v/v lactic acid<br>(230 mL) | 300 W<br>Xe lamp<br>$\lambda > 400$ nm | 26 | 5.7%<br>(420 nm) | [4] |
| $(CdS)_{0.4}$/$(ZnS)_{0.6}$ | 50 mg catalyst<br>0.1 M $Na_2S$ &<br>0.1 M $Na_2SO_3$<br>(100 mL) | 300 W<br>Xe lamp<br>$\lambda \geq 420$ nm | 0.83 | - | [5] |
| Zn/Cd-MOF derived<br>ZnS/CdS heterojunction | 20 mg catalyst<br>35 mM $Na_2S$ &<br>18 mM $Na_2SO_3$<br>(100 mL) | 300 W<br>Xe lamp<br>$\lambda \geq 420$ nm | 2.35 | 3.92%<br>(420 nm) | [6] |
| Pt-PdS-CdS/ZnS<br>core/shell NCs | 1.5 mg catalyst<br>0.1 M $Na_2S$<br>(50 mL) | 300 W<br>Xe lamp<br>$\lambda \geq 380$ nm | ~50 | - | [7] |
| PdS-CdS/ZnS<br>core/shell particles | 100 mg catalyst<br>0.1 M $Na_2S$ &<br>0.1 M $Na_2SO_3$<br>(270 mL) | 300 W<br>Xe lamp<br>$\lambda \geq 400$ nm | 2.08 | - | [8] |
| CdS/ZnS core/shell<br>nanorods | 10 mg catalyst<br>0.5 M $Na_2S$ &<br>0.5 M $Na_2SO_3$<br>(100 mL) | 300 W<br>Xe lamp<br>AM 1.5G | 24.1 | 9.3%<br>(420 nm) | [9] |
| CdS/ZnS core/shell<br>particles | 5 mg catalyst<br>0.1 M $Na_2S$ &<br>0.1 M $Na_2SO_3$<br>(50 mL) | 20 W<br>LED<br>$\lambda = 420$ nm | 16 | 8.78%<br>(420 nm) | [10] |
| CdS/ZnS-NiS<br>microstructures | 10 mg catalyst<br>0.35 M $Na_2S$ &<br>0.25 M $Na_2SO_3$<br>(25 mL) | 225 W<br>Xe lamp<br>$\lambda \geq 320$ nm | 60.44 | 23.3%<br>(365 nm) | [11] |
| ZnS-CuS-CdS<br>particles | 100 mg catalyst<br>0.35 M $Na_2S$ &<br>0.25 M $Na_2SO_3$<br>(150 mL) | 150 W<br>Xe lamp<br>AM 1.5G | 0.84 | - | [12] |
| $Cd_{0.5}Zn_{0.5}S$ nano-twins | 100 mg catalyst<br>0.35 M $Na_2S$ &<br>0.25 M $Na_2SO_3$<br>(180 mL) | 300 W<br>Xe lamp<br>$\lambda > 430$ nm | 17.9 | 43%<br>(425 nm) | [13] |

| | | | | | | |
|---|---|---|---|---|---|---|
| $Cd_{0.5}Zn_{0.5}S$ twined nanorods | 100 mg catalyst 0.35 M $Na_2S$ & 0.25 M $Na_2SO_3$ (180 mL) | 300 W Xe lamp $\lambda > 430$ nm | 25.8 | 62% (425 nm) | [14] |
| **50-ZnS/CdS NCAs** | **20 mg catalyst 1.4 M $Na_2S$ & 1.0 M $Na_2SO_3$ (20 mL)** | **300 W Xe lamp $\lambda \geq 420$ nm** | **29.0** | **60% (420 nm)** | **This work** |

**Table S3.** EIS equivalent circuit fitted parameters for the mesoporous CdS and ZnS NCAs and the as-prepared ZnS/CdS materials.

| **Sample** | **$R_s$ (Ohm)** | **$C_{dl}$ (F)** | **$R_{ct}$ (Ohm)** | **$[x^2]$[1]** |
|---|---|---|---|---|
| CdS NCAs | 14.2 | $63.3 \times 10^{-6}$ | 1312 | $2.1 \times 10^{-4}$ |
| 10-ZnS/CdS | 12.7 | $24.2 \times 10^{-5}$ | 686 | $2.3 \times 10^{-4}$ |
| 30-ZnS/CdS | 17.4 | $17.9 \times 10^{-5}$ | 474 | $4.3 \times 10^{-4}$ |
| 50-ZnS/CdS | 12.1 | $19.5 \times 10^{-5}$ | 285 | $8.7 \times 10^{-4}$ |
| 70-ZnS/CdS | 13.4 | $76.8 \times 10^{-6}$ | 486 | $2.0 \times 10^{-4}$ |
| 90-ZnS/CdS | 12.3 | $86.5 \times 10^{-6}$ | 747 | $4.2 \times 10^{-4}$ |
| ZnS NCAs | 18.0 | $11.4 \times 10^{-5}$ | 1362 | $2.5 \times 10^{-4}$ |
| ZnS/CdS bulk [2] | 9.6 | $62.5 \times 10^{-6}$ | 1499 | $10.3 \times 10^{-4}$ |

[1] In EIS analysis, the fitting error is often expressed using the chi-square metric. Typically, a satisfactory fit corresponds to chi-square values ranging from $10^{-2}$ to $10^{-4}$. [2] EIS results for the ZnS/CdS bulk reference catalyst (50 wt.% ZnS).

**Table S4**. PL lifetime biexponential decay model fitting parameters and calculated average lifetimes for the mesoporous CdS and 50-ZnS/CdS NCAs, and the ZnS/CdS bulk analogue with 50 wt.% ZnS.

| **Catalyst** | **$A_1$ (%)** | **$\tau_1$ (ns)** | **$A_2$ (%)** | **$\tau_2$ (ns)** | **$\tau_{av}$ (ns)** |
|---|---|---|---|---|---|
| CdS NCAs | 72.5 | 0.80 ± 0.006 | 27.5 | 4.35 ± 0.039 | 3.20 ± 0.017 |
| 50-ZnS/CdS | 65.0 | 1.00 ± 0.012 | 35.0 | 4.73 ± 0.055 | 3.70 ± 0.025 |
| ZnS/CdS bulk | 68.5 | 0.70 ± 0.005 | 31.5 | 4.08 ± 0.026 | 3.16 ± 0.011 |

[1]The average lifetime ($\tau_{av}$) was calculated by the equation: $\tau_{av} = (\sum_i A_i \tau_i^2)/(\sum_i A_i \tau_i)$ (i = 1, 2).

## Supporting Figures

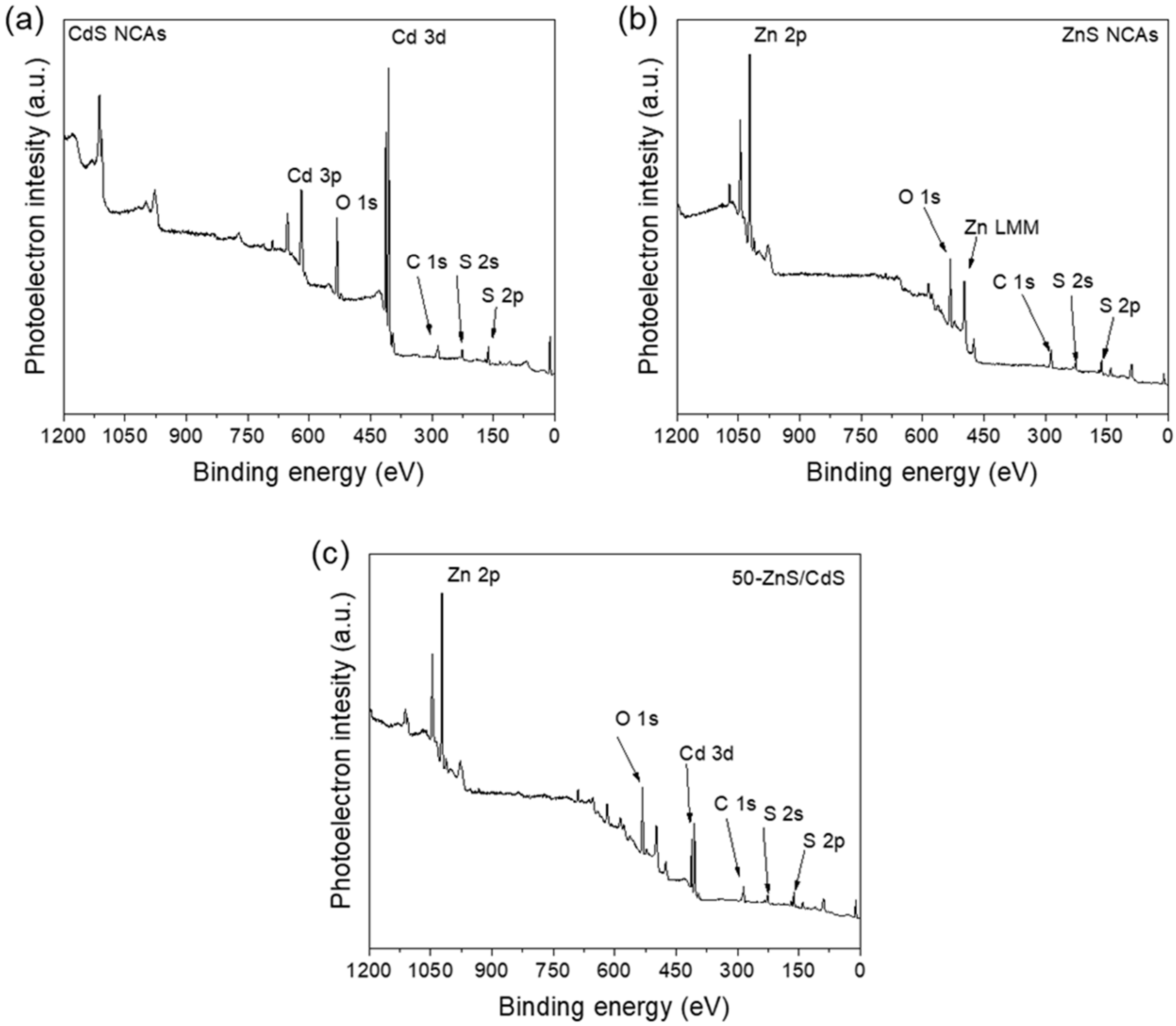


**Figure S1.** Typical XPS survey spectra of CdS, ZnS and 50-ZnS/CdS NCAs showing the presence of Cd, Zn and S, from the constituent NCs and C and O from adsorbed molecules.

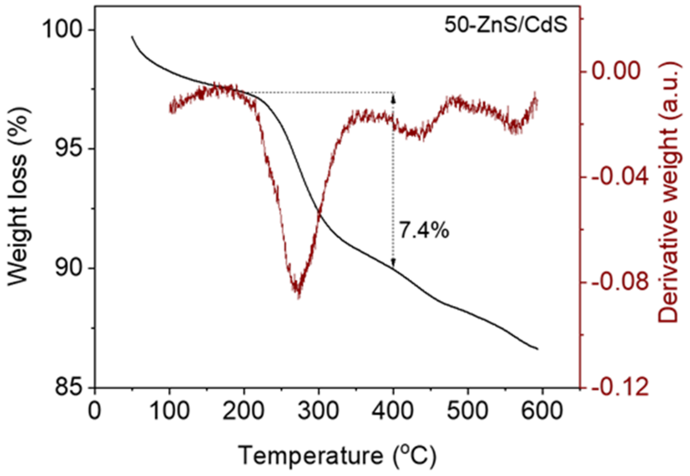


**Figure S2**. Example of TGA profile for the mesoporous (black line) 50-ZnS/CdS nanocomposite obtained after three cycles of ethanol/water washing process. The weight loss in this sample indicated by the corresponding differential thermogravimetric (DTG) curve (red line) is attributed to the decomposition of organic matter.

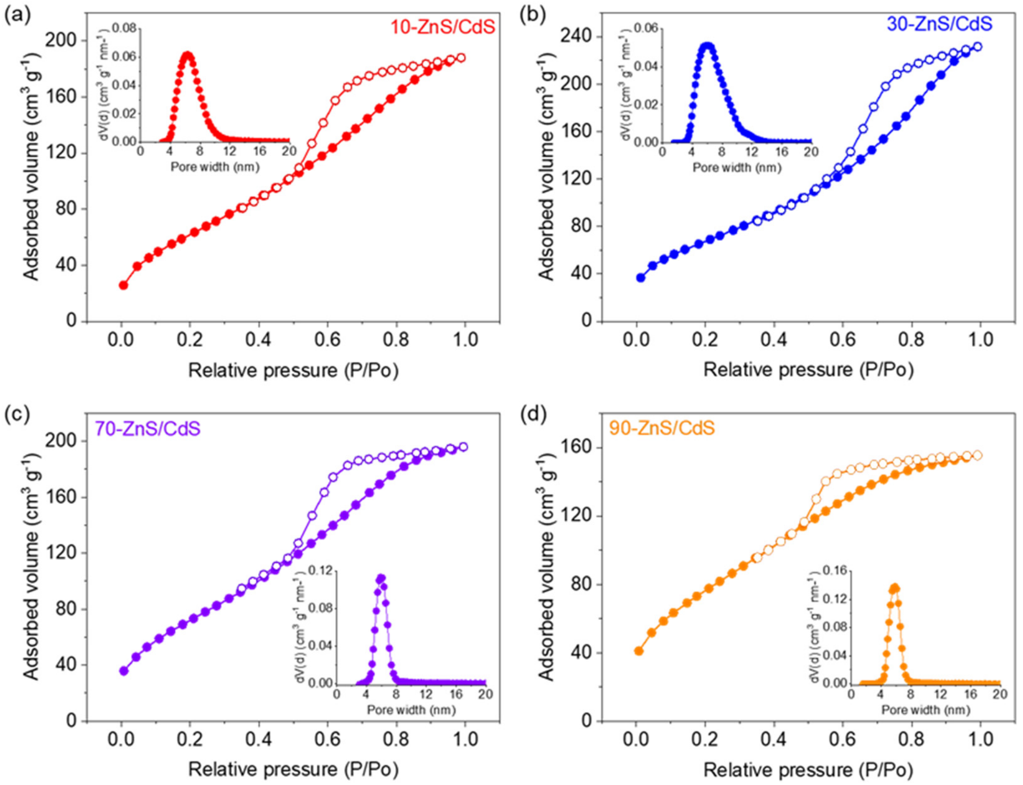


**Figure S3**. Nitrogen adsorption (filled cycles) and desorption (open cycles) isotherms at −196 °C for the mesoporous ZnS/CdS nanocomposites featuring different ZnS loadings: (**a**) 10 wt.%, (**b**) 30 wt.%, (**c**) 70 wt.%, and (**d**) 90 wt.%. Insets: The corresponding NLDFT pore size distributions calculated from the adsorption branch of isotherms.

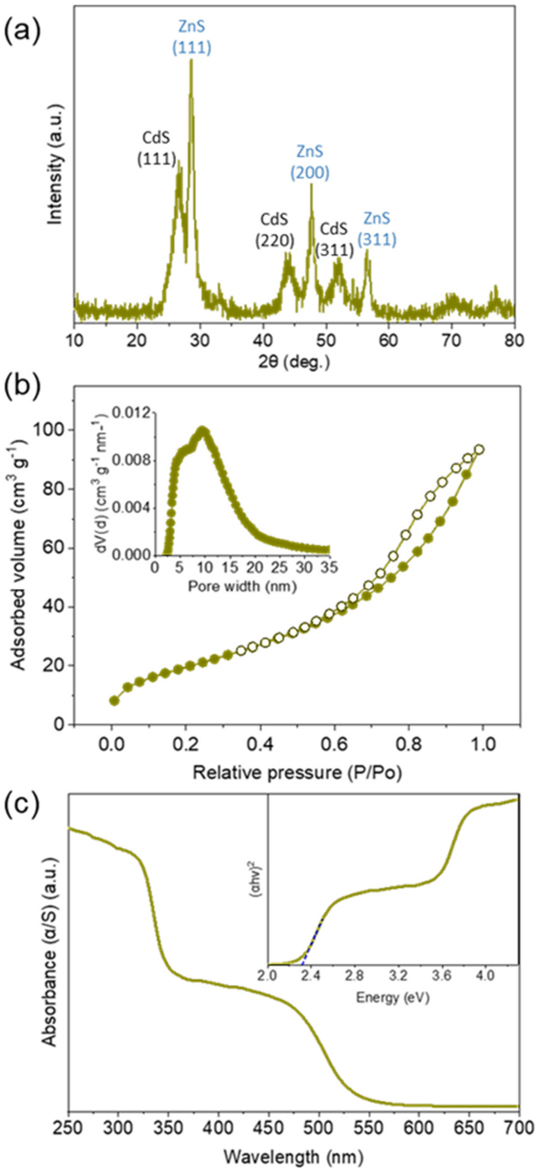


**Figure S4.** (**a**) XRD pattern, (**b**) $N_2$ adsorption (filled cycles) and desorption (open cycles) isotherms at –196 °C and the corresponding NLDFT pore-size distribution plot (inset), and (**d**) optical absorption spectrum and Tauc plot (inset) of the reference ZnS/CdS bulk catalyst with 50 wt.% ZnS content.

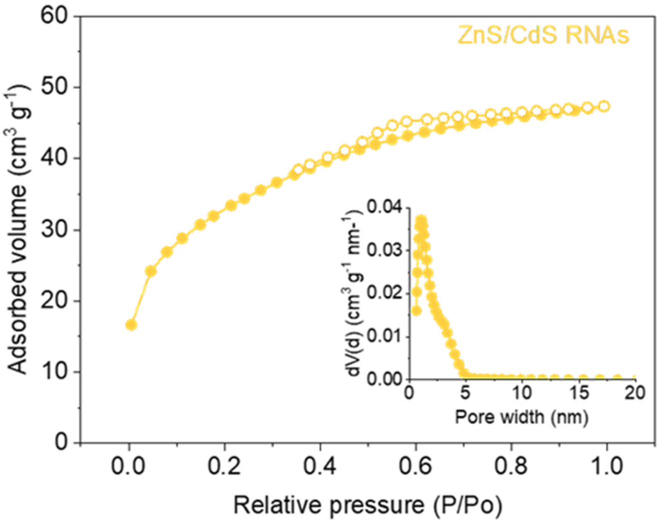


**Figure S5**. $N_2$ adsorption (filled cycles) and desorption (open cycles) isotherms at –196 °C and the corresponding NLDFT pore-size distribution plot (inset) of the reference ZnS/CdS RNAs catalyst with 50 wt.% ZnS content. Analysis of the adsorption data with the BET method gives a surface area of 114 $m^2$ $g^{-1}$ and total pore volume of 0.07 $cm^3$ $g^{-1}$. The NLDFT pore size distribution calculated from the adsorption branch of isotherms indicates a pore size of ~ 1.5 nm.

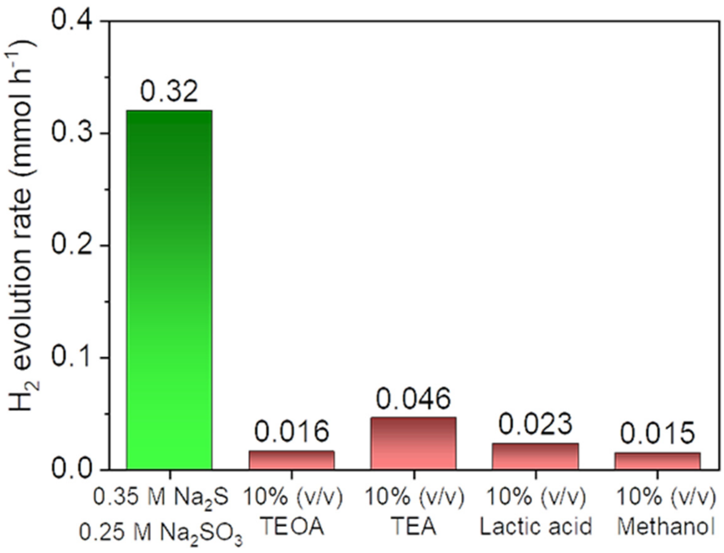


**Figure S6**. Photocatalytic $H_2$ evolution rates (averaged over 3-h reaction period) for the mesoporous 50-ZnS/CdS catalyst using different sacrificial reagents: 0.35 M $Na_2S$ and 0.25 M $Na_2SO_3$, triethanolamine (TEOA, 10% v/v), triethylamine (TEA, 10% v/v), lactic acid (10% v/v) and methanol (10% v/v). All photocatalytic reactions were performed as follows: 20 mg of catalyst dispersed in a 20 mL aqueous solution containing the sacrificial reagent; 300 W Xe light irradiation with a long-pass cut-off filter allowing $\lambda \geq 420$ nm.

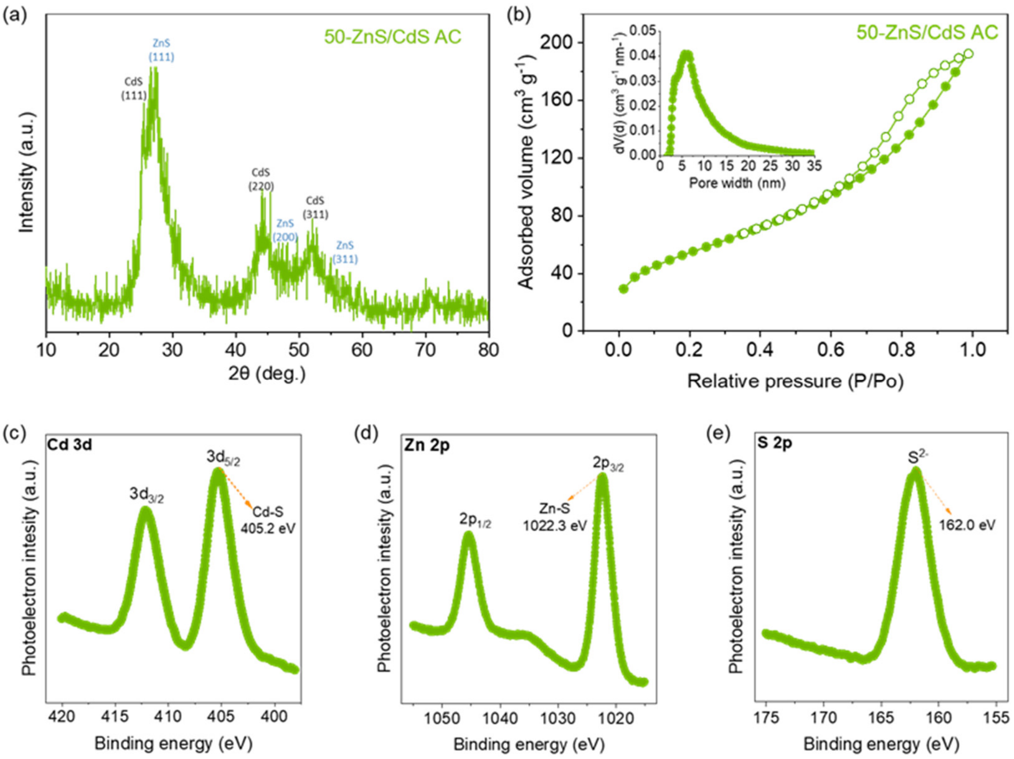


**Figure S7**. (**a**) XRD pattern, (**b**) $N_2$ adsorption (filled cycles) and desorption (open cycles) isotherms at –196 °C (inset: the corresponding NLDFT pore-size distribution plot), and (**c-e**) typical XPS spectra of the (**c**) Cd 3d, (**d**) Zn 2p, and (**e**) S 2p regions of the 50-ZnS/CdS catalyst retrieved after 24-h of photocatalytic reaction.

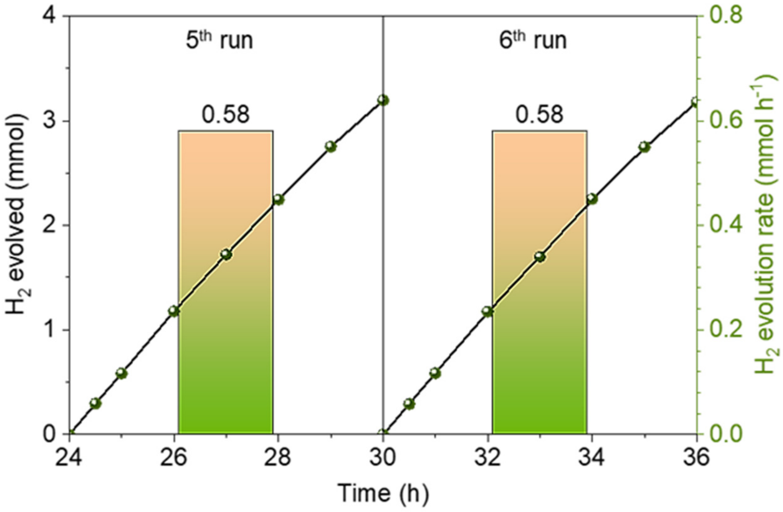


**Figure S8.** 5th and 6th run of the photocatalytic recycling study of the 50-ZnS/CdS catalyst in a 1.4 M $Na_2S$ and 1.0 M $Na_2SO_3$ aqueous electrolyte. All the $H_2$-evolution rates obtained as an average over the initial 3-h reaction period. Reaction conditions: 20 mg catalyst dispersed in 20 mL of fresh electrolyte solution, 300 W Xenon light irradiation with a UV cutoff filter (λ ≥ 420 nm), T = 20 ± 2 °C.

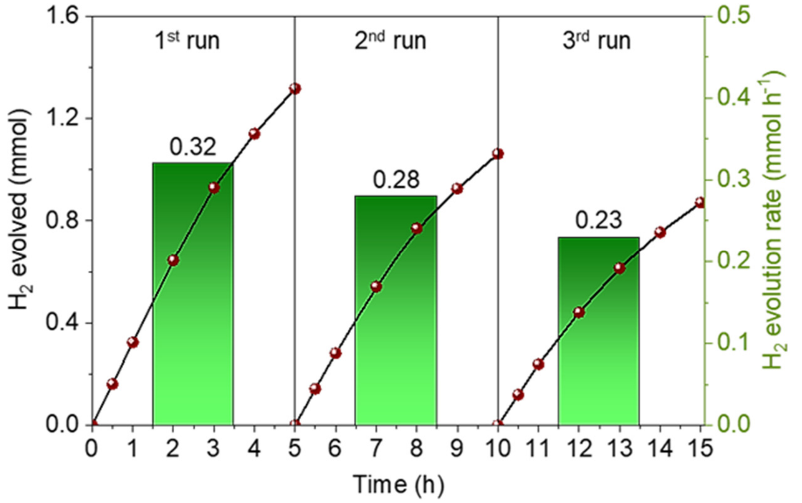


**Figure S9.** Photocatalytic recycling tests of the 50-ZnS/CdS catalyst in a 0.35 M $Na_2S$ and 0.25 M $Na_2SO_3$ aqueous electrolyte. All the $H_2$-evolution rates obtained as an average over the initial 3-h reaction period. Reaction conditions: 20 mg catalyst dispersed in 20 mL of fresh electrolyte solution, 300 W Xenon light irradiation with a UV cutoff filter ($\lambda \geq 420$ nm), T = 20 ± 2 °C.

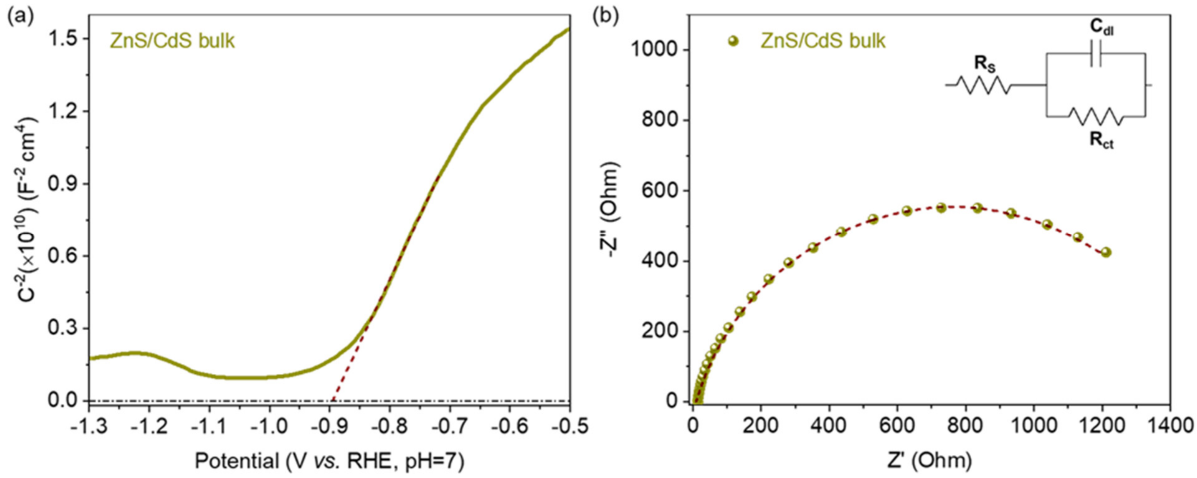


**Figure S10**. (**a**) Mott-Schottky plot and (**b**) EIS Nyquist spectrum (inset: equivalent circuit model) for the reference ZnS/CdS bulk catalyst with 50 wt.% ZnS content.

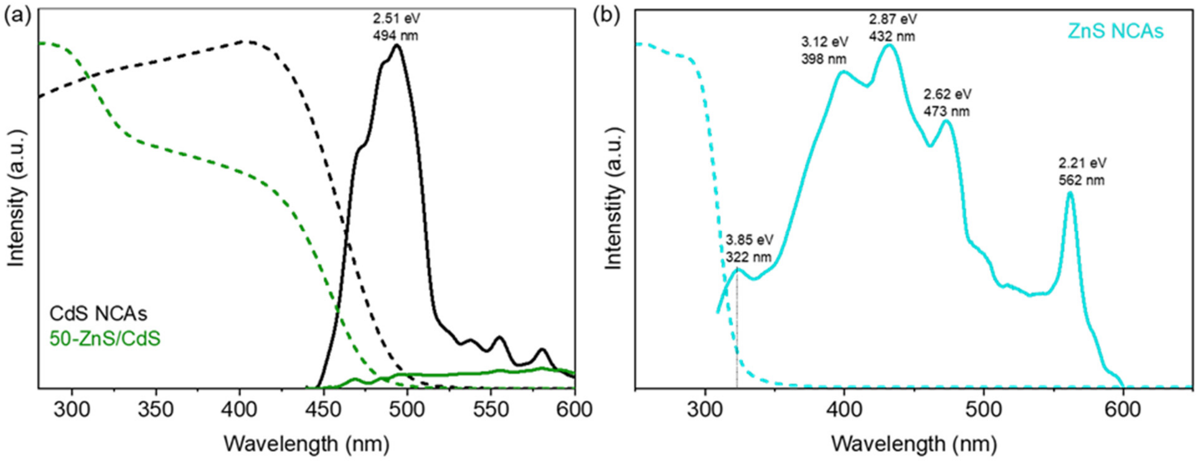


**Figure S11**. Room-temperature PL emission spectra (solid lines) of (**a**) mesoporous CdS and 50-ZnS/CdS NCAs (excitation wavelength 380 nm) and (**b**) mesoporous ZnS NCAs (excitation wavelength 280 nm). The dashed lines correspond to the UV-Vis/NIR absorption spectra of the respective samples.

## References


1. Li, K.; Chen, R.; Li, S.L.; Han, M.; Xie, S.L.; Bao, J.C.; Dai, Z.H.; Lan, Y.Q. Self-Assembly of a Mesoporous ZnS/Mediating Interface/CdS Heterostructure with Enhanced Visible-Light Hydrogen-Production Activity and Excellent Stability. *Chem Sci* **2015**, *6*, 5263–5268, doi:10.1039/C5SC01586C.

2. Jiang, D.; Sun, Z.; Jia, H.; Lu, D.; Du, P. A Cocatalyst-Free CdS Nanorod/ZnS Nanoparticle Composite for High-Performance Visible-Light-Driven Hydrogen Production from Water. *J Mater Chem A Mater* **2015**, *4*, 675–683, doi:10.1039/C5TA07420G.

3. Wang, X.; Li, X. yan Heterostruture CdS/ZnS Nanoparticles as a Visible Light-Driven Photocatalyst for Hydrogen Generation from Water. *Int J Green Energy* **2016**, *13*, 1201–1208, doi:10.1080/15435075.2016.1175349/SUPPL_FILE/LJGE_A_1175349_SM6365.DOC.

4. Wang, J.; Wang, Z.; Li, L.; Chen, J.; Zheng, J.; Jia, S.; Zhu, Z. Structure-Controlled CdS(0D, 1D, 2D) Embedded onto 2D ZnS Porous Nanosheets for Highly Efficient Photocatalytic Hydrogen Generation. *RSC Adv* **2017**, *7*, 24864–24869, doi:10.1039/C7RA02565C.

5. Kundu, J.; Satpathy, B.K.; Pradhan, D. Composition-Controlled CdS/ZnS Heterostructure Nanocomposites for Efficient Visible Light Photocatalytic Hydrogen Generation. *Ind Eng Chem Res* **2019**, *58*, 22709–22717, doi:10.1021/ACS.IECR.9B03764/ASSET/IMAGES/LARGE/IE9B03764_0001.JPEG.

6. Zhu, Y.; Jiang, X.; Lin, L.; Wang, S.; Chen, C. Fabrication of ZnS/CdS Heterojunction by Using Bimetallic MOFs Template for Photocatalytic Hydrogen Generation. *Chem Res Chin Univ* **2020**, *36*, 1032–1038, doi:10.1007/S40242-020-0083-5/METRICS.

7. Huang, L.; Wang, X.; Yang, J.; Liu, G.; Han, J.; Li, C. Dual Cocatalysts Loaded Type i CdS/ZnS Core/Shell Nanocrystals as Effective and Stable Photocatalysts for H2 Evolution. *Journal of Physical Chemistry C* **2013**, *117*, 11584–11591, doi:10.1021/JP400010Z/SUPPL_FILE/JP400010Z_SI_001.PDF.

8. Xie, Y.P.; Yu, Z.B.; Liu, G.; Ma, X.L.; Cheng, H.M. CdS–Mesoporous ZnS Core–Shell Particles for Efficient and Stable Photocatalytic Hydrogen Evolution under Visible Light. *Energy Environ Sci* **2014**, *7*, 1895–1901, doi:10.1039/C3EE43750G.

9. Lin, Y.; Zhang, Q.; Li, Y.; Liu, Y.; Xu, K.; Huang, J.; Zhou, X.; Peng, F. The Evolution from a Typical Type-I CdS/ZnS to Type-II and Z-Scheme Hybrid Structure for Efficient and Stable Hydrogen Production under Visible Light. *ACS Sustain Chem Eng* **2020**, *8*, 4537–4546, doi:10.1021/ACSSUSCHEMENG.0C00101/ASSET/IMAGES/LARGE/SC0C00101_0003.JPEG.

10. Reddy, N.L.; Rao, V.N.; Kumari, M.M.; Sathish, M.; Muthukonda Venkatakrishnan, S. Development of High Quantum Efficiency CdS/ZnS Core/Shell Structured Photocatalyst for the Enhanced Solar Hydrogen Evolution. *Int J Hydrogen Energy* **2018**, *43*, 22315–22328, doi:10.1016/J.IJHYDENE.2018.10.054.

11. Ma, D.; Shi, J.W.; Sun, L.; Sun, Y.; Mao, S.; Pu, Z.; He, C.; Zhang, Y.; He, D.; Wang, H.; et al. Knack behind the High Performance CdS/ZnS-NiS Nanocomposites: Optimizing Synergistic Effect

between Cocatalyst and Heterostructure for Boosting Hydrogen Evolution. *Chemical Engineering Journal* **2022**, *431*, 133446, doi:10.1016/J.CEJ.2021.133446.

12. Hong, E.; Kim, D.; Kim, J.H. Heterostructured Metal Sulfide (ZnS–CuS–CdS) Photocatalyst for High Electron Utilization in Hydrogen Production from Solar Water Splitting. *Journal of Industrial and Engineering Chemistry* **2014**, *20*, 3869–3874, doi:10.1016/J.JIEC.2013.12.092.

13. Liu, M.; Wang, L.; Lu, G.; Yao, X.; Guo, L. Twins in Cd1−xZnxS Solid Solution: Highly Efficient Photocatalyst for Hydrogen Generation from Water. *Energy Environ Sci* **2011**, *4*, 1372–1378, doi:10.1039/C0EE00604A.

14. Liu, M.; Jing, D.; Zhou, Z.; Guo, L. Twin-Induced One-Dimensional Homojunctions Yield High Quantum Efficiency for Solar Hydrogen Generation. *Nature Communications 2013 4:1* **2013**, *4*, 1–8, doi:10.1038/ncomms3278.
